# A review of *Lista* Walker, 1859 in China, with descriptions of five new species (Lepidoptera, Pyralidae, Epipaschiinae)

**DOI:** 10.3897/zookeys.642.7157

**Published:** 2017-01-03

**Authors:** Mingqiang Wang, Fuqiang Chen, Chunsheng Wu

**Affiliations:** 1 Key Laboratory of Zoological Systematics and Evolution, Institute of Zoology, Chinese Academy of Sciences, Beijing 100101, P.R. China; 2 University of Chinese Academy of Sciences, Beijing, 100049, P. R. China

**Keywords:** China, new record, new species, Pyraloidea, taxonomy

## Abstract

Ten species of the genus *Lista* are recognized from China. Among them, five species are described as new to science, namely, *Lista angustusa* **sp. n.**, *Lista gilvasa* **sp. n.**, *Lista longifundamena* **sp. n.**, *Lista menghaiensis* **sp. n.**, and *Lista sichuanensis* **sp. n.** Diagnoses are provided for the genus and five previously described species, *Lista haraldusalis* (Walker, 1859), *Lista insulsalis* (Lederer, 1863), *Lista ficki* (Christoph, 1881), *Lista plinthochroa* (West, 1931), and *Lista variegata* (Moore, 1888), that occur in China. Two species, *Lista plinthochroa* and *Lista variegata*, are reported from China for the first time. All adults and their genital structures are illustrated. A key to the Chinese species is provided.

## Introduction

The genus *Lista* was erected by Walker in 1859, based on *Lista genisusalis* Walker, 1859 as the type species. Subsequently Lederer (1863) erected *Paracme* for *insulsalis* (Lederer, 1863) from Ningbo (Zhejiang, China), Christoph (1881) erected *Craneophora* for *ficki* (Christoph, 1881) from Heilongjiang (China), and Butler (1889) proposed *Belonepholis* for the species *striata* from Dharmsala (India) as a junior synonym of *ficki*. These genera were synonymized with *Lista* by Solis (1992), a paper which also listed seven species under *Lista* by Solis (1992), *Lista carniola* (Hampson, 1916), *Lista ficki*, *Lista haraldusalis* (Walker, 1859), *Lista insulsalis*, *Lista sumatrana* (Hering, 1901), *Lista variegata* (Moore, 1888) and *Lista plinthochroa* (West, 1931). Yamanaka described a new species, *Lista monticola* Yamanaka, 2000, from Nepal. At present, eight species of *Lista* have been reported all over the world and are distributed in the Palaearctic and Oriental Regions.

In China, three species were previously recorded, *Lista ficki*, *Lista insulsalis* and *Lista haraldusalis* (Caradja 1925; Wang et al. 2003; Li and Ren 2008). In this paper, two species, *Lista plinthochroa* and *Lista variegata*, are newly recorded from China, and an additional five species are described as new to science.

## Material and methods

The specimens examined and the types of the new species are deposited in the collection of the Institute of Zoology, Chinese Academy of Sciences (IZCAS), Beijing, P. R. China. The specimens were collected with different methods, but mainly by light traps. The photographs of moths and their genitalia were taken with a NIKON D7000 digital camera connected to a NIKON SMZ 1500 stereomicroscope. Methods of dissection, morphometrics, and terminology follow Wang et al. (2003) and Slamka (2006).

## Taxonomic account

### Lista

Taxon classificationAnimaliaLepidopteraPyralidae

Walker, 1859

Lista Walker, 1859: 877. Type species: Lista genisusalis Walker, 1859
Paracme Lederer, 1863: 338. Type species: Poracme insulsalis Lederer, 1863
Craneophora Christoph, 1881: 1. Type species: Craneophora ficki Christoph, 1881
Belonepholis Butler, 1889: 89. Type species: Belonepholis striata Butler, 1889

#### Diagnosis.

The genus is very special in its external characters. It can be easily distinguished from other genera of the subfamily by having a much brighter and conspicuous wing pattern. The valva usually has spines or sclerotized plate medially located in the male genitalia and the oval or rounded corpus bursae have the rounded signa in the female genitalia, which are same as in the genus *Stericta*. But it differs from the latter by the shapes of valva and juxta. In general, the wings have an orange to yellow postmedial fascia with dark brown edges. In the male genitalia, the uncus usually has long or short spines laterally located, the sacculus always has two sclerotized processes medially located, and the valva usually has a variously-shaped sclerotized plate in the central area distal to the saccular processes.

#### Description.

Head covered with dense scales; labial and maxillary palpi upturned; antennae filiform, male with a scape extension covered with dense scales. Both wings with similar patterns, fasciae indistinct except postmedial fascia, postmedial fasciae conspicuous, and smooth at border.

#### Male genitalia.

Uncus broad, gnathos various. Valva broad, and outer margin usually truncated; costa sclerotized; sacculus well developed, often with hook-like or spine-like processes, usually extending backward to base of valva. Phallus slender, slightly curved.

#### Female genitalia.

Ovipositor covered with dense setae. Sterigma associated with the ostium bursae appears to be sclerotized. The papillae analis are not extruded and are located within the 8^th^ segment; the sterigma is lightly sclerotized and the lamella postvaginalis is variously sclerotized. Apophysis anterior nearly same length or longer than apophysis posterior. Ductus bursae slender, usually membranous. Corpus bursae elliptic or rounded, usually with two rounded or oval-shaped signa consisting of many minute spines.

#### Distribution.

China, Russia, Korea, Japan, India, Nepal, Sri Lanka, Vietnam, Myanmar, Malaysia, Indonesia, Philippines, New Guinea.

#### Key to the species of *Lista* in China

**Table:** 

1	Uncus with two spines	**2**
–	Uncus without spines	**6**
2	Uncus with spines laterally located, hindwing with pink-fuscous scales	**3**
–	Uncus with spines medially located, hindwing with pale-yellow scales	***Lista variegata***
3	Spines nearly as long as uncus; forewing covered with more fuscous scales than yellow	***Lista insulsalis***
–	Spines half as long as uncus; forewing with more yellow scales than fuscous	**4**
4	Juxta with spines about 1/2 of length of juxta at apex	***Lista longifundamena* sp. n.**
–	Juxta with spines about 1/3 of length of juxta at apex	**5**
5	Base of juxta broad, postmedial fascia slightly curved, ductus bursae as same as in width	***Lista haraldusalis***
–	Base of juxta narrow, postmedial fascia straight, lower ductus bursae broader than upper	***Lista gilvasa* sp. n.**
6	Gnathos with two spines	**7**
–	Gnathos without spines	**8**
7	Middle of sacculus with a serrated sclerotized plate, bent towards outer margin of plate and with a short thorn-like process	***Lista angustusa* sp. n.**
–	Middle of sacculus with a spine-like process, bent towards outside	***Lista sichuanensis* sp. n.**
8	Juxta with apex rounded	**9**
–	Juxta with apex pointed	***Lista ficki***
9	Male with long hair scales nearly 2/3 of abdomen length at abdomen end. Middle of sacculus with a long spine and a short thorn-like process, Top of ductus bursae slightly sclerotized	***Lista plinthochroa***
–	Male with short hair scales nearly 1/4 of abdomen at abdomen end, middle of sacculus with a long sclerotized plate and a short thorn-like processes, the sclerotized plate with apex serrated, ductus bursae membranous	***Lista menghaiensis* sp. n.**

### Lista angustusa sp. n.

Taxon classificationAnimaliaLepidopteraPyralidae

http://zoobank.org/12F9A4BB-E225-442F-BE21-2104F358766D

Figs 1 , 13 , 23

#### Diagnosis.

The new species is very similar to *Lista haraldusalis* in wing pattern, but the wing color of the new species is darker than the latter. In the male genitalia, it can be distinguished from the latter by the narrower valva, having two lateral spines in the gnathos, and lacking spines in the uncus.

**Figures 1–6.**
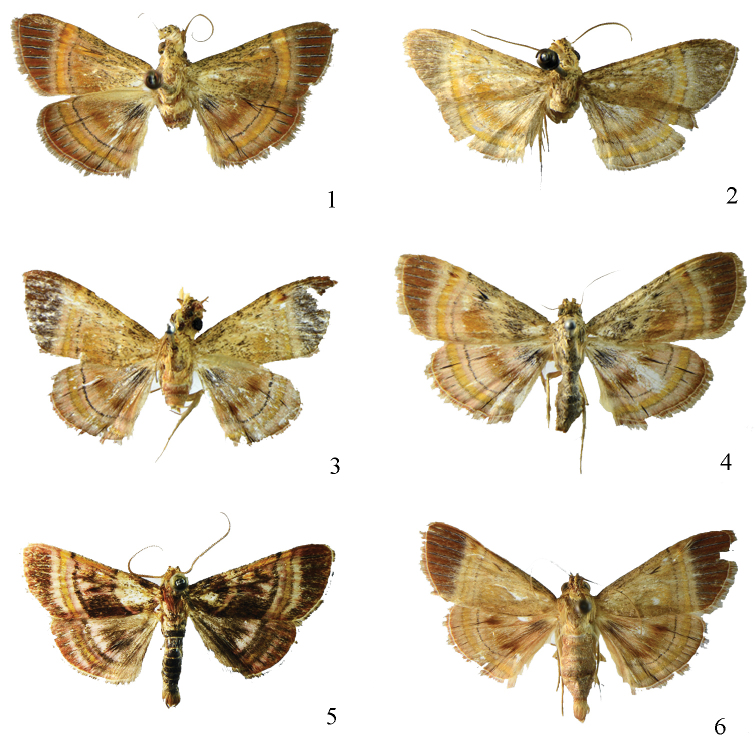
Adults. **1** *Lista angustusa* sp. n., male, holotype **2** *Lista ficki* (Christoph, 1881), male **3** *Lista gilvasa* sp. n., male, holotype **4** *Lista haraldusalis* (Walker, 1859), female **5** *Lista insulsalis* (Lederer, 1863), male **6** *Lista longifundamena* sp. n., male, holotype.

#### Description.

Adult. Forewing length 8.5–11.0 mm (*n* = 7). Head pale yellow, mixed with brown; labial palpus upturned, third segment pointed; antenna brown, scape extension black, with pale grey scales on inner side, and blackish-brown on outer side. Thorax mixed with pale yellow, blackish-brown and pale grey scales. Forewing covered with brown, yellow and pink scales; base mixed with yellow and black scales; postmedial fascia orange with dark brown edges, outer area covered with fuscous and dark pink scales; cilia brown. Hindwing with same pattern as forewing.

**Male genitalia** (Fig. 13). Uncus broad, densely suffused with setae, apex truncated. Gnathos incurved apically, with three spines at apex and a long spine laterally located. Valva nearly the same width from base to apex, apex obliquely truncated; costa obviously sclerotized, apex swollen; sacculus expanded at base, with two processes in middle, the inner one larger, with serrated edge, the outer one small thorn-like, a sclerotized plate from sacculus to valva medially located. Juxta constricted and bifurcated, two pointed plates at apex. Phallus cylindrical, curved slightly.

**Female genitalia** (Fig. 23). Ovipositor nearly round, suffused with setae. Apophysis anterior and apophysis posterior nearly same length. Ductus bursae short, membranous. Corpus bursae elliptic, with two rounded signa.

#### Holotype.

♂, Jiangxi: Jiulianshan, 11.VI.1975, Song Shimei (gen. slide. no. Ep540).

#### Paratypes.

5♀♀, locality and collector same as holotype, 21.VI–8.VIII.1975, (gen. slide. no. Ep562). Guangdong: Ruyuan, Nanling, 1♂, 20.VII.2008, Chen Fuqiang (gen. slide. no. Ep549); Chebaling, 1♂, 22.VII.2008, Chen Fuqiang (gen. slide. no. Ep543).

#### Distribution.

China (Jiangxi, Guangdong).

#### Etymology.

The specific name is derived from Latin *angustus* (= narrow) in accordance with its narrow valva.

### Lista ficki

Taxon classificationAnimaliaLepidopteraPyralidae

(Christoph, 1881)

Figs 2 , 14 , 24

Craneophora ficki Christoph, 1881: 2; Janse 1931: 439–491; Inoue 1982: 379, pl. 45, fig. 40; Yamanaka and Yoshiyashu 1992: 91.
Belenopholis striata Butler, 1889: 90, pl. 134, f. 3.
Lista ficki (Christoph): Solis 1992: 283.

#### Diagnosis.

The species is different from congeners by paler scales on the forewing, the tip of the gnathos without denticulation, and the juxta swollen, the apex bifurcated with two pointed processes.

#### Material examined.

Guangdong: Dinghushan, 1♀, 7.VII.1973, Li Tiesheng. Jiangxi: Jiulianshan, 1♂, 23.IX.1979, Song Shimei. Hubei: Shennongjia, 3♂♂1♀, 950–1250m, 3–16.VII.1980, Yu Peiyu & Han Yinheng (gen. slide no. Ep552); Xingshan, 1♂3♀♀, 1350m, 18.VII.1993, Song Shimei (gen. slide no. Ep532). Gansu: Wenxian, 1♀, 720m, 28.VII.1999, Yao Jian; Zhouqu, 1♀, 2400m, 14.VII.1999, Zhu Chaodong.

#### Distribution.

China (Heilongjiang, Gansu, Hubei, Jiangxi, Guangdong, Guangxi); India, Japan, Philippines.

#### Remarks.

Yamanaka (2000) reported this species in Nepal. However, according to the related literature, the genitalia are quite different from those of *Lista ficki* (Christoph, 1881) provided by Janse (1931). Meanwhile, their structure of the male genitalia matches the figure of *Lista haraldusalis* (Walker, 1859) provided by Marumo (1942). Therefore, his reported species actually is *Lista haraldusalis* (Walker, 1859) rather than *Lista ficki* (Christoph, 1881).

### Lista gilvasa sp. n.

Taxon classificationAnimaliaLepidopteraPyralidae

http://zoobank.org/AB832BCC-54D7-4147-BC89-20B306FE65A6

Figs 3 , 15 , 25

#### Diagnosis.

The new species is similar to *Lista longifundamena* sp. n. Both species are different from other congeners by the straight postmedial fascia on the forewing; however, *Lista longifundamena* sp. n. has the valva slightly constricted from the middle to the apex, the sacculus with the inner processes at middle more slender, and the apex of the juxta bifurcated with two strongly sclerotized slender arms.

#### Description.

Adult. Forewing length 9.5–10.5mm (*n* = 4). Head yellow; labial palpus upturned, mixed with yellow and black scales; maxillary palpus pale yellow; antenna pale brown, scape extension black, mixed with golden scales in male. Thorax mixed with blackish-brown and a small number of yellow scales. Forewing covered with brown, yellow, black and pink scales; postmedial fascia straight, pale yellow with brown edges, outer area covered with fuscous and dark pink scales; cilia brown. Hindwing with similar pattern to forewing, but more brown scales than forewing in central area, and outer area covered with pink scales.

**Male genitalia** (Fig. 15). Uncus broad, densely covered with setae, two spine-like processes at base laterally. Gnathos narrower and more sclerotized than uncus. Valva slightly broader from base to apex, apex rounded; costa slightly sclerotized, apex swollen; sacculus with two processes in middle, the inner one spine-like, the outer one thorn-like, a thin sclerotized plate from sacculus to center of valva. Juxta peltate and bifurcated, apex pointed. Phallus curving slightly, with a crescent-shaped cornutus at apex.

**Female genitalia** (Fig. 25). Ovipositor slightly narrow, suffused with setae. Apophysis anterioris longer than apophysis posterioris. Ductus bursae slender, membranous. Corpus bursae round, with two signa, nearly oval.

#### Holotype.

♂, Guangxi: Napobeidou, 550m, 22.VI.2000, Zhu Chaodong (gen. slide no. Ep524).

#### Paratayes.

Guangxi: Longzhou, 1♂, 550m, 22.VI.1963, Wang Chunguang (gen. slide no. Ep529); Jinxiu, Luoxiang, 1♀, 200m, 15.V.1999, Han Hongxiang; Shangsi, 1♀, 300m, 29.V.1999, Zhang Xuezhong (gen. slide no. Ep563).

#### Distribution.

China (Guangxi).

#### Etymology.

The specific name is derived from Latin *gilvas* (= pale yellow), in accordance with the yellow postmedial fascia of the new species.

### Lista haraldusalis

Taxon classificationAnimaliaLepidopteraPyralidae

(Walker, 1859)

Figs 4 , 16 , 26

Locastra haraldusalis Walker, 1859: 160.
Lista genisusalis Walker, 1859: 877.
Stericta haraldusalis (Walker): Hampson 1896: 121; Lu and Guan 1953: 109.
Craneophora haraldusalis (Walker): Janse 1931: 473–474.
Lista haraldusalis (Walker): Solis 1992: 283.
Lista ficki (nec. Christoph): Yamanaka 2000: 67–69 (misidentified).

#### Diagnosis.

The species can be distinguished from other species of the genus by its specific gnathos. In this species, the gnathos is broader and more sclerotized than that in other species and it has a serrated apex that is located medially.

#### Material examined.

Hubei: Shennongjia, 2♂♂, 950m, 3–17.VII.1980, Yu Peiyu; Shennongjia, 1♀, 500m, 30.V.1981, Han Yinheng; Shennongjia, 2♂♂4♀♀, 500–1250m, 16.VI–4.VII.1981, Han Yinheng (gen. slide no. Ep556); Shennongjia, 2♂♂2♀♀, 860–920m, 28.VII–17.VIII.1981, Han Yinheng; Xingshan, 3♂♂, 1350m, 18.VII.1993, Song Shimei (gen. slide no. Ep75, Ep527); Digui, 5♂♂5♀♀, 110–117m, 3–6.IX.1994, Han Yinheng (gen. slide no. Ep73, Ep526, Ep551, Ep74, Ep522). Shaanxi: Zhenba, 1♀, 5.VI.1981; Zhouzhi, 2♀♀, 1350m, 24.VI.1999, Zhu Chaodong; Foping, 1♀, 900m, 27.VI.1999, Zhang Youwei (gen. slide no. Ep516); Foping, 4♂♂6♀♀, 867m, 15.VIII.2007, Li Wenzhu (gen. slide no. Ep515). Zhejiang: Linan, 3♀♀, 1350m, 28–29.VII.2003, Xue Dayong & Han Hongxiang (gen. slide no. Ep517). Gansu: Wenxian, 2♂♂, 720–1000m, 20–28.VII.1999, Yao Jian (gen. slide no. Ep541). Guangxi: Longsheng, 1♀, 11.VI.1980, Xue Dayong; Napo, Baihe, 2♀♀, 440m, 6–7.IV.1998, Wu Chunsheng & Li Wenzhu (gen. slide no. Ep557); Jinxiu, 1♀, 300m, 29.IV.1999, Yao Jian. Yunnan: Pingbian, 1♀, 1500m, 19.VI.1956, Huang Keren; Menghai, Xishuangbanna, 1♀, 1200–1600m, 20.VII.1958, Pu Fuji; Xishuangbanna, 1♂, 650m, 25.VII.1962, Song Shimei; Da Menglong, Xishuangbanna, 1♀, 650m, 29.V.1962, Song Shimei; Xishuangbanna, 1♀, 15.V.1978, Wang Shuyong; Weixi, 1♀, 2500m, 25.VII.1981, Wang Shuyong; Lufeng, 1♀, 23.VI.1982; Daguan, 1♂1♀, 780m, 1.VII.1982, Luo Feijin (gen. slide no. Ep559); Nabanhe, 1♀, 1083m, 25.VI.2014, Liu Xiuwei (gen. slide no. Ep566). Anhui: Jiuhuashan, 1♂, 1979m, 24.VII.2003 (gen. slide no. Ep565). Guizhou: Congjiang, 5♂♂1♀, 1–10.VIII.2013, Yang Maofa (gen. slide no. Ep567). Fujian: Sangang, 1♀, 740m, 30.VI.1960, Zhang Yiran; Sangang, 1♀, 18.IX.1979, Yu Chunren; Wuyishan, 1♀, 650m, 27.V.2000, Wang Jiashe; Wuyishan, 1♀, 650m, 27.VII.2000, Song Shimei; Xianfengling, 1♀, 500m, 4.VI.1981, Qi Shicheng. Sichuan: Emeishan, 1♀, 800–1000m, 13.VI.1957, Zhu Fuxing. Xizang: Motuo, 1♂1♀, 1.VI.1983, Han Yinheng. Hainan: Jianfengling, 1♀, 26.X.1982; Wuzhishan, 1♂, 9.XII.2007, Chen Fuqiang (gen. slide no. Ep577).

#### Distribution.

China (Shaanxi, Gansu, Anhui, Zhejiang, Hubei, Jiangxi, Fujian, Hainan, Guangxi, Sichuan, Guizhou, Yunnan, Xizang), India, Japan, Nepal, Malaysia.

#### Remarks.

Li and Ren (2008) reported this species in Henan; however, the genitalia are quite different from those of *Lista haraldusalis* (Walker, 1859) provided by Marumo (1942). Meanwhile, their figure of the adult matches the original figure of *Lista insulsalis* (Lederer, 1863). Thus, their description actually refers to *Lista insulsalis* (Lederer, 1863) rather than *Lista haraldusalis* (Walker, 1859). In addition, the species was wrongly recognized as *Lista ficki* by Yamanaka (2000). We correct their identification here.

### Lista insulsalis

Taxon classificationAnimaliaLepidopteraPyralidae

(Lederer, 1863)

Figs 5 , 17 , 27

Paracme insulsalis Lederer, 1863: 339, pl. 6. f. 11.
Stericta rubiginetincta Caradja, 1925: 58; Lu and Guan 1953: 109.
Lista insulsalis (Lederer): Solis 1992: 283.
Lista haraldusalis (nec. Walker): Li and Ren 2008: 35 (misidentified).

#### Diagnosis.

The species differs from other species by rustier colored scales on the wings, the uncus with two extremely elongated arms laterally located, and the slender processes of the sacculus about twice as long as in other species.

#### Material examined.

Hunan: Nanyue, 2♂♂8♀♀, 29.V.1974, Song Shimei (gen. slide no. Ep13, Ep22); Hengshan, 1♂7♀♀, 29.V.1974, Song Shimei; Hengshan, 2♂♂1♀, 16.VIII.1979, Zhang Baolin. Fujian: Wuyishan, 2♀♀, 12–17.VIII.1979, Song Shimei; Yezhou, 1♀, 13.V.1980; Jiangle, 2♀, 16.IX.1990; Wuyishan, 8♂♂2♀♀, 520–1260m, 24–30.VII.2000, Song Shimei & Wang Jiashe. Zhejiang: Huangyan, 1♀, 26.IX.1962, Zhang Baolin; Tianmushan, 3♂♂2♀♀, 1–2.IX.1981, Song Shimei (gen. slide no. Ep71); Lin’an, 8♂♂6♀♀, 1350m, 28–29.VII.2003, Xue Dayong & Han Hongxiang (gen. slide no. Ep510, Ep511, Ep512); Anji, 1♂1♀, 13.VII.1995, Wu Hong & Wang Zhengru; 1♀, 23.VII.1996; Shaanxi: Luonan, 1♀, 8.IX.1980; Liuba, 2♂♂3♀♀, 1350m, 19–24.VII.1998, Yao Jian & Zhang Xuezhong (gen. slide no. Ep513); Foping, 2♀♀, 950m, 23–24.VII.1998, Yao Jian & Yuan Decheng; Ningshan, 1♂, 1580m, 27.VII.1998, Yuan Decheng. Henan: Xinyang, 1♂, 250m, 20–21.VII.2002, Han Hongxiang. Guangdong: Dinghushan, 1♂, 29.VII.2005, Chen Fuqiang (gen. slide no. Ep541); Ruyuan, Nanling, 2♂♂, 865m, 15.VII.2005, Chen Fuqiang; Lianping, 1♀, 13.V.1973, Zhang Baolin. Jiangxi: Guling, 2♀♀, 30.VII–VIII.1935; Jiulianshan, 1♀, 11.VI.1975, Song Shimei; Lushan, 4♀♀, 16–30.VII.1980, Song Shimei; Luzhi, 1♂2♀♀, 30.VI–9.VII.1980, Shanxi: Zhongtiaoshan, 4♀♀, VIII.1978, Zhu Huiqian. Jiangsu: 2♀♀, 21–26.VIII.1933. Anhui: Huangshan, 1♂, 29.VII.1976. Yunnan: Xishuangbanna, 1♂, 1200–1600m, 18.VII.1958, Wang Shuyong; Luxi, 2♂♂1♀, 7–8.V.1980, Song Shimei; Yiliang, 8♂♂1♀, 19–20.VII.1982, Song Shimei; Lufeng, 2♂♂, 22.VI.1982, Song Shimei; Yongsheng, 1♂, 2250m, 10.VII.1984, Liu Dajun; Shiping, 1♂, 1650m, Liu Yongjie. Xinjiang: Urumqi, 1♀, 5.VI.1984; Guangxi: Xing’an, 2♀♀, 5.VI.1984; Miaoershan, 1♂, 8.VII.1985; Shangsi, 3♂♂4♀♀, 250–300m, 27–29.V.1999, Yuan Decheng et al.; Jinxiu, 1♂2♀♀, 200–900m, 20–29.V.1999, Li Wenzhu et al.; Shangsi, 1♀, 250–300m, 9.VI.2000, Zhu Chaodong.

#### Distribution.

China (Hebei, Shanxi, Henan, Shaanxi, Gansu, Xinjiang, Jiangsu, Anhui, Zhejiang, Hubei, Jiangxi, Hunan, Fujian, Taiwan, Guangdong, Hainan, Guangxi, Sichuan, Guizhou, Yunnan), Russia, Korea, India, Sri Lanka, Myanmar, Indonesia.

#### Remarks.

The species was wrongly recognized as *Lista haraldusalis* (Walker, 1859) by Li and Ren (2008). We correct their identification here.

### Lista longifundamena sp. n.

Taxon classificationAnimaliaLepidopteraPyralidae

http://zoobank.org/B225247C-D569-44A9-A60D-A7D5054B6AD0

Figs 6 , 18 , 28

#### Diagnosis.

This new species is larger in body than other species of the genus. The species is similar to *Lista gilvasa* sp. n. Their differences are described under *Lista gilvasa* sp. n.

#### Description.

Adult. Forewing length 11.5–13.0mm (*n* = 6). Head pale brown, mixed with fuscous scales; labial palpus upturned, mixed with fuscous scales; maxillary palpus pale yellow; antenna brown, scape extension fuscous, mixed with golden scales in male. Thorax mixed with pale brown and black scales. Forewing covered with pale brown, yellow and pink scales; postmedial fascia straight, yellow with brown edges, outer area covered with fuscous and dark pink scales; cilia brown. Hindwing with similar pattern as forewing, but basal area mixed with black scales and outer area covered with pink scales.

**Male genitalia** (Fig. 18). Uncus broad, suffused with dense setae, two spine-like processes at base. Gnathos incurved apically, with four small spines at apex. Valva slightly constricted from middle to apex, terminal margin truncated; costa slightly sclerotized, sacculus with two processes in middle, the inner one spine-like, slender, the outer one with mini spines at apex, a thin sclerotized plate from sacculus to centre of valva. Juxta bifurcated at apex and with two strongly sclerotized slender arms at both sides. Phallus slender, curved in middle, with a sclerotized plate-like cornutus.

**Female genitalia** (Fig. 28). Ovipositor broad, suffused with setae. Apophysis anterioris 1/3 longer than apophysis posterioris. Ductus bursae slender, membranous. Corpus bursae elliptic, with two slightly sclerotized of rounded signa.

#### Holotype.

♂, Hainan: Jianfengling, 18–20.IV.1982, Chen Zhiqin (gen. slide. no. Ep531).

#### Paratypes.

Hainan: 1♀, 16.III.1982, Zhang Baolin (gen. slide. no. Ep555); Jianfengling, 2♂♂2♀♀, 4.XI.1981, Liu Yuanfu (gen. slide. no. Ep122, Ep561); Jianfengling, 1♀, 18–20.IV.1982, Chen Zhiqin; Jianfengling, 1♂, 4.XI.1981 (gen. slide. no. Ep122).

#### Distribution.

China (Hainan).

#### Etymology.

The name is derived from Latin *longus* (= long) and *fundamen* (= base), in accordance with the juxta and two long spines at base.

### Lista menghaiensis sp. n.

Taxon classificationAnimaliaLepidopteraPyralidae

http://zoobank.org/76C7A60E-5A2F-46D8-9542-0492D1A76518

Figs 7 , 19 , 29

#### Diagnosis.

This new species is very similar to *Lista haraldusalis*, but the basal area on the forewing is slightly paler than the latter. In the male genitalia, the gnathos has three spines at the apex in the new species while the gnathos has a serrated apex in *Lista haraldusalis*. Furthermore, the sclerotized plate on the valva strongly extends toward the outer margin in the new species.

**Figures 7–12.**
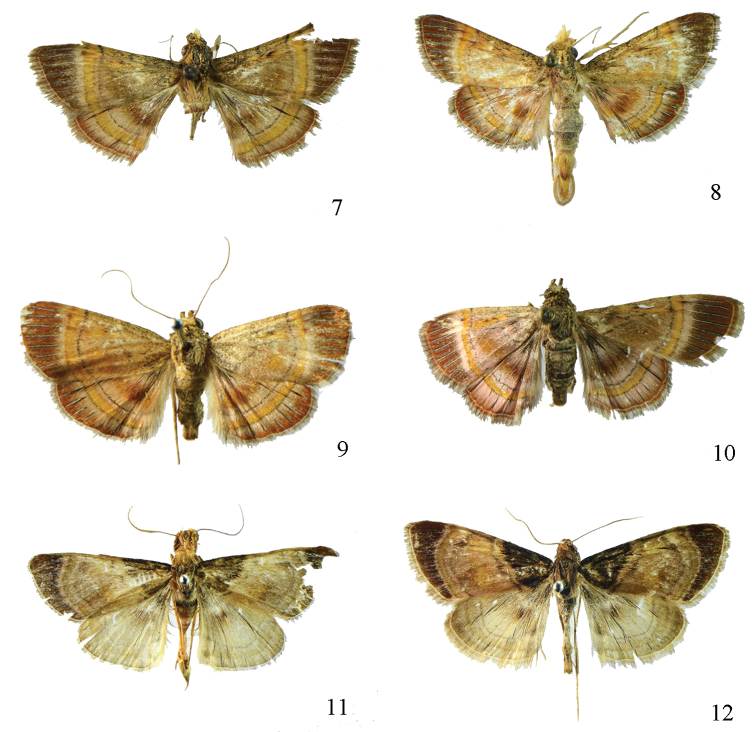
Adults. **7** *Lista menghaiensis* sp. n., male, holotype **8** *Lista plinthochroa* (West, 1931), male **9** *Lista plinthochroa* (West, 1931), female **10** *Lista sichuanensis* sp. n., female, paratype **11** *Lista variegata* (Moore, 1888), male **12** *Lista variegata* (Moore, 1888), female.

#### Description.

Adults. Forewing length 9.0–10.5mm (*n* = 2). Head yellow, mixed with blackish-brown; labial palpus upturned, mixed with pale yellow and black scales; maxillary palpus pale yellow; antenna brown; scape extension black, mixed with yellow scales in male. Thorax mixed with brown and fuscous scales. Forewing covered with brown, yellow or pink scales; basal area mixed with yellow and pale brown scales; costal margin with two black spots at middle and terminal trisection; postmedial fascia yellow with brown edges, outer area covered with dark pink scales; cilia brown. Hindwing with same pattern as forewing.

**Male genitalia** (Fig. 19). Uncus broad, suffused with dense setae totally. Gnathos with three spines at apex and two long spines 1/3 of gnathos at base medially. Valva broad, with terminal smoothly incurved; sacculus with two processes in middle, the inner one strong with apex serrated, the outer one thorn-like; a well-developed sclerotized plate from sacculus to center of valva, strongly extended toward outer manrgin. Juxta bifurcated, blunt. Phallus slightly curved.

**Figures 13–18.**
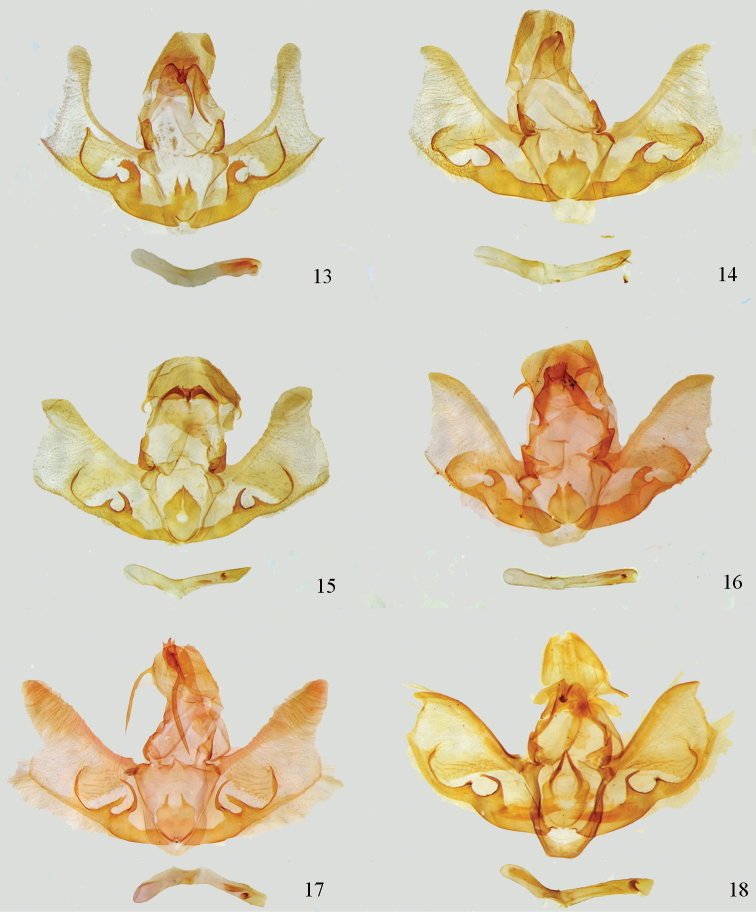
Male genitalia. **13** *Lista angustusa* sp. n., holotype, gen. slide no. Ep549 **14** *Lista ficki* (Christoph, 1881), gen. slide no. Ep523 **15** *Lista gilvasa* sp. n., holotype, gen. slide no. Ep524 **16** *Lista haraldusalis* (Walker, 1859), gen. slide no. Ep527 **17** *Lista insulsalis* (Lederer, 1863), gen. slide no. Ep511 **18** *Lista longifundamena* sp. n., holotype, gen. slide no. Ep122.

**Figures 19–22.**
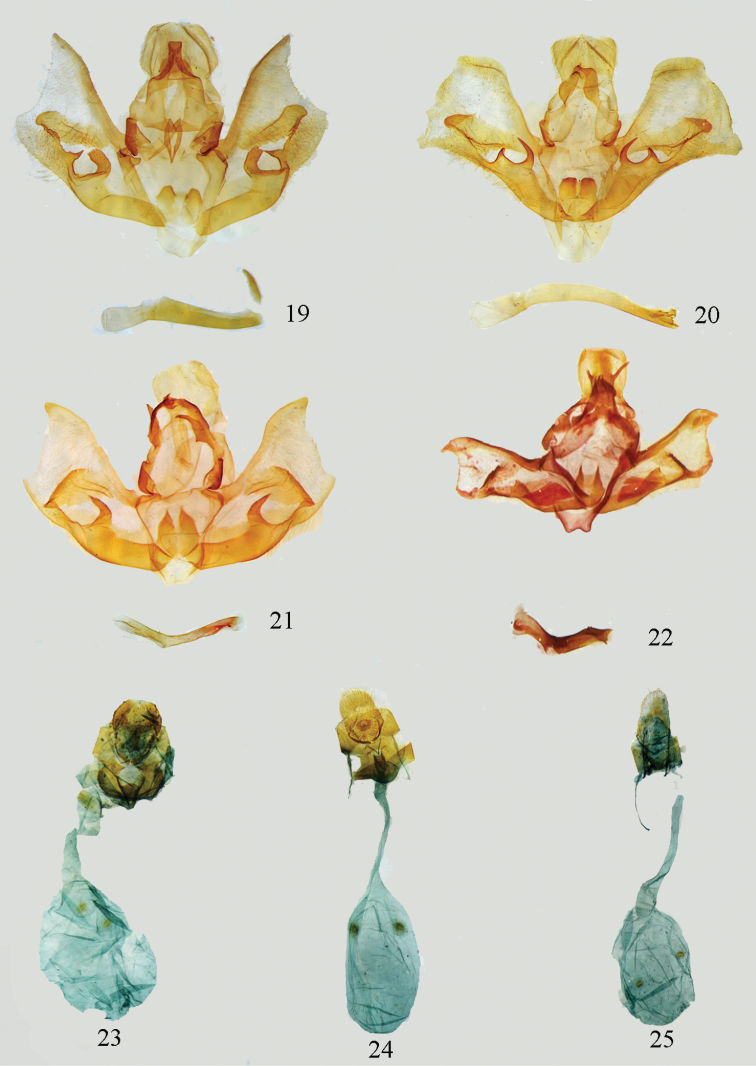
Male genitalia. **19** *Lista menghaiensis* sp. n., holotype, gen. slide no. Ep539 **20** *Lista plinthochroa* (West, 1931), gen. slide no. Ep543 **21** *L .sichuanensis* sp. n., paratype, gen. slide no. Ep521 **22** *Lista variegata* (Moore, 1888), gen. slide no. Ep123.

**Female genitalia** (Fig. 29). Ovipositor broad, suffused with setae. Apophysis anterioris 1/4 longer than apophysis posterioris. Antrum incurved at apex. Ductus bursae slender, membranous. Corpus bursae elliptic, with two signa, slightly rounded sclerotized.

**Figures 23–32.**
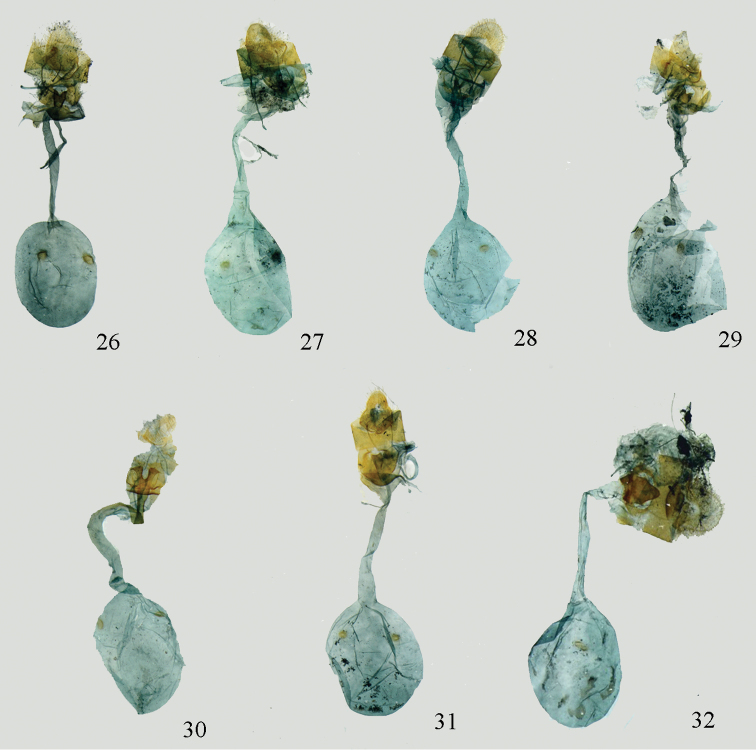
Female genitalia. **23** *Lista angustusa* sp. n., paratype, gen. slide no. Ep562 **24** *Lista ficki* (Christoph, 1881), gen. slide no. Ep552 **25** *Lista gilvasa* sp. n., paratype, gen. slide no. Ep563 **26** *Lista haraldusalis* (Walker, 1859), gen. slide no. Ep557 **27** *Lista insulsalis* (Lederer, 1863), gen. slide no. Ep512 **28** *Lista longifundamena* sp. n., paratype, gen. slide no. Ep555 **29** *Lista menghaiensis* sp. n., paratype, gen. slide no. Ep558 **30** *Lista plinthochroa* (West, 1931), gen. slide no. Ep553 **31** *Lista sichuanensis* sp. n., paratype, gen. slide no. Ep554 **32** *Lista variegata* (Moore, 1888), gen. slide no. Ep603.

#### Holotype.

♂, Yunnan: Xishuangbanna, Menghai, 18.VII.1958, Wang Shuyong (gen. slide. no. Ep539).

#### Paratype.

1♀, same data as holotype (gen. slide no. Ep558).

#### Distribution.

China (Yunnan).

#### Etymology.

The species is named after the type locality, Menghai in Yunnan province.

### Lista plinthochroa

Taxon classificationAnimaliaLepidopteraPyralidae

(West, 1931)

Figs 8–9 , 20 , 30

Stericta plinthochroa West, 1931: 210.
Lista plinthochroa (West): Solis 1992: 283.

#### Diagnosis.

This species is significantly different from others by the end of the abdomen with long hair-like scales. In the male genitalia, the valva broadens from the base to the outer margin and the sclerotized plate on the valva extends toward outer margin.

#### Material examined.

Sichuan: Emeishan, 3♂♂16♀♀, 800–1000m, 6.V–30.VI.1957, Zhu Fuxing & Huang Keren (gen. slide no. Ep11, Ep518, Ep525, Ep6–1, Ep572); Emeishan, 1♀, 9.VI.1979, Gao Ping; Qinchengshan, 1♂, 700–1600m, 30.V.1979 (gen. slide no. Ep520); Fujian: Sangang, Wuyishan, 1♂, 1.VI.1983, Mai Guoqing; Sangang, 2♀♀, 740m, 6–17.VII.1980, Zhang Yiran; Sangang, 1♀, 740m, 26.IV.2000, Wang Jiashe. Yunnan: Pingbian, 1♀, 1500m, 8.VI.1956, Huang Keren. Gansu: Chengxian, 1♂, 1020m, 4.VII.1999, Yaojian; Kangxian, 1♂, 1400m, 8.VII.1999, Zhu Chaodong (gen. slide no. Ep528). Taiwan: Pinglin, 1♀, 1000m, 13.IV.1984; Lianhuachi, 1♀, 11.VIII.1984, Wang Xiaoyue. Guangdong: Nanling, 1♂1♀, 865m, 9–15.VII.2005, Chen Fuqiang. Jiangxi: Jiulianshan, 1♂6♀♀, 21.VI–30.VII.1975, Song Shimei; Dayu, 13♂♂7♀♀, 14.VI–16.VIII.1975 (gen. slide no. Ep519, Ep553). Hainan: Jianfengling, 3♂♂, 26.III.1984, Song Shimei (gen. slide no. Ep543, Ep574, Ep576).

#### Distribution.

China (Gansu, Jiangxi, Fujian, Taiwan, Guangdong, Hainan, Sichuan, Yunnan), Philippines.

#### Remarks.

The species is reported in China for the first time.

### Lista sichuanensis sp. n.

Taxon classificationAnimaliaLepidopteraPyralidae

http://zoobank.org/E3FB5DA9-91CA-4A47-BA7C-88D8702B513E

Figs 10 , 21 , 31

#### Diagnosis.

This species is hard to distinguish by its external characters. It differs from other species by the sacculus with a single process in the middle, while the sacculus usually has two processes in other species. Furthermore, the sclerotized plate on the valva is twice or three times as broad as others.

#### Description.

Adults. Forewing length 11.0–12.5mm (*n* = 6). Head blackish-brown; labial palpus upturned, mixed with fuscous and black scales, stronger in male; maxillary palpus pale yellow; antenna brown, scape extension black, mixed with fuscous scales in male. Thorax mixed with brown and fuscous scales. Forewing covered with brown, black and pink scales; base mixed with yellow and black scales; costal margin yellow; postmedial fascia orange with brown edges, outer area covered with pink and fuscous scales; cilia grey. Hindwing with same pattern as forewing.

**Male genitalia** (Fig. 21). Uncus broad, suffused with dense setae. Gnathos broad, base extended to two strongly sclerotized spine-like processes laterally located, apex with three spines. Valva nearly the same width from base to apex; costa extruded the apex of valva; sacculus with a single spine-like process in middle; a well-developed sclerotized broad plate from sacculus to centre of valva. Juxta bifurcated with two pointed plates at apex. Phallus slender, slightly curved.

**Female genitalia** (Fig. 31). Ovipositor broad, densely suffused with setae. Apophysis anterioris longer than apophysis posterioris. Ductus bursae slender, membranous. Corpus bursae round, with two rounded signa, slightly sclerotized.

#### Holotype.

♂, Sichuan: Dukou, 14.VI.1981 (gen. slide. no. Ep521).

#### Parataypes.

Sichuan: Qingchenshan, 1♂, 5.VI.1979, Shang Jinwen; same data as holotype, 2♂♂2♀♀ (gen. slide. no. Ep554).

#### Distribution.

China (Sichuan).

#### Etymology.

The species is named after the type locality, Sichuan Province.

### Lista variegata

Taxon classificationAnimaliaLepidopteraPyralidae

(Moore, 1888)

Figs 11–12 , 22 , 32

Scopocera variegata Moore, 1888: 203, pl. 7. f. 4.
Lista variegata (Moore): Solis 1992: 283.

#### Diagnosis.

The species differs from others by the hindwing with pale yellow scales. In the male genitalia, the center of the uncus has two spines, and the phallus is about half as long as others.

#### Material examined.

Xizang: Nielamu, Zhangmu, 1♂, 2232m, 12.V.1974, Zhang Xuezhong; Motuo, Gedang, 1♀, 4.IX.1982, Lin Zai; Bomi, 1♂, 2700m, 5.IX.1983, Han Yinheng (gen. slido no. Ep123); Linzhi, Niyanghe, 2♂♂, 3000m, 2.VIII.2006, Chen Fuqiang; Milin, Paixiang, 2♀♀, 2910m, 5.VIII.2006, Chen Fuqiang; Bomi, Zhamuzhen, 2♀♀, 2840m, 28.VIII.2006, Chen Fuqiang (gen. slide no. Ep603).

#### Distribution.

China (Xizang), India.

#### Remarks.

The species is reported in China for the first time.

## Supplementary Material

XML Treatment for Lista

XML Treatment for Lista angustusa

XML Treatment for Lista ficki

XML Treatment for Lista gilvasa

XML Treatment for Lista haraldusalis

XML Treatment for Lista insulsalis

XML Treatment for Lista longifundamena

XML Treatment for Lista menghaiensis

XML Treatment for Lista plinthochroa

XML Treatment for Lista sichuanensis

XML Treatment for Lista variegata
